# Case Report: Ultrasonography and Magnetic Resonance Imaging of Anterior Segment Dysgenesis in a Calf

**DOI:** 10.3389/fvets.2022.794255

**Published:** 2022-04-08

**Authors:** Takeshi Tsuka, Yoshiharu Okamoto, Yuji Sunden, Takehito Morita, Takao Amaha, Norihiko Ito, Yusuke Murahata, Masamichi Yamashita, Tomohiro Osaki, Tomohiro Imagawa

**Affiliations:** Department of Veterinary Clinical Medicine, Faculty of Agriculture, School of Veterinary Medicine, Tottori University, Tottori, Japan

**Keywords:** anterior segment dysgenesis, calf, enucleation, exophthalmos, magnetic resonance imaging, ultrasonography

## Abstract

This study includes diagnostic efficacy of the antemortem, combined use of ultrasonography and magnetic resonance imaging (MRI) for the diagnosis of anterior segment dysgenesis. A 7-day-old male Holstein calf presented with progressive unilateral exophthalmos associated with enlargement of the right eyeball soon after birth. Ultrasonography of the enlarged right eyeball showed (1) a 2-cm-thick echogenic parenchymal lesion filling the anterior region of the right eyeball, (2) excess accumulation of the anechoic vitreous humor, and (3) absence of the lens structure. Antemortem examination using T2-weighted and fluid-attenuated inversion recovery MRI revealed a thickened, hyperintense anterior lesion and absence of the lens structure. These imaging findings were suggestive of anterior segment dysgenesis. Antemortem imaging showed no abnormalities other than the abnormal structure and size of the right eyeball; therefore, enucleation of the right eye was performed, which allowed intact healing without suppuration. Ocular ultrasonography enhanced the diagnostic accuracy due to the characteristic ultrasonographic findings of a thickened anterior lesion and absence of the lens structure in the eyeball, suggestive of anterior segment dysgenesis.

## Introduction

Anterior segment dysgenesis (ASD) is a rare congenital ocular disease, which occurs as a result of the abnormal maturation of neuro-ectodermal cells during the organogenetic period of the eye (1). ASD is a common cause of unilateral or bilateral exophthalmos (2–4). Besides an ASD, multiple bovine diseases associated with involvements of exophthalmos are previously reported, including systemic or local infections of *Moraxella* spp. (5), bovine leukosis virus (BLV) (6, 7), and *Theileria annulata* (8), cavernous sinus syndrome (9), intraocular or extraorbital formation of tumors and abscesses (10–12), and hereditary ocular diseases (13, 14).

Antemortem observation of morphological abnormalities inside the affected eyeballs of bovine exophthalmos cases (other than the enlarged size) may assist in differential diagnosis. In the veterinary field, imaging modalities such as ultrasonography, computed tomography (CT), and magnetic resonance imaging (MRI) can be used to observe the eyes and diagnose ocular diseases (15, 16). All three of these imaging modalities have been previously used in practice with bovines, although MRI was applied for the necropsy of a skull (10, 12, 17). The purpose of the present study was to evaluate the clinical applicability of ultrasonography and MRI for diagnosis and therapeutic planning in a calf with ASD. In addition, these findings were compared with previous human and bovine reports.

## Case Presentation

**Figure 1:**
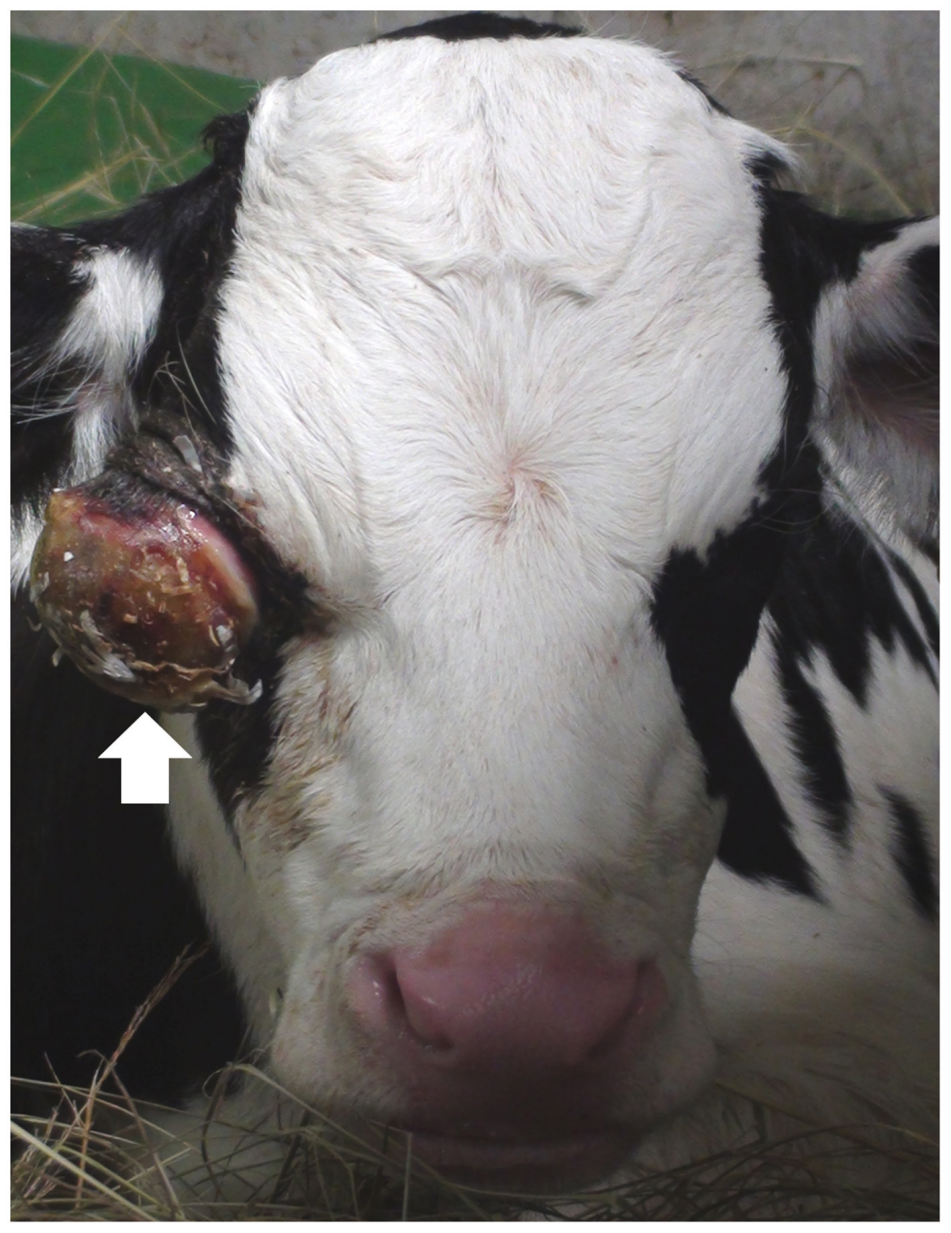
Photo of the front of the face of the 7-day-old male Holstein calf, showing the enlarged eyeball (arrow) protruding from the right eye-socket.

A 7-day-old male Holstein calf presented with progressive exophthalmos of the right eye, soon after birth (Figure 1). The eyeball protruded more than half-way out of the right eye socket and enlarged by ~5 cm during the first 3 days after birth. The sides of the protruding right eyeball were soft, but most of the anterior surface was dry and hard. The similar ocular abnormality has never been found in the animals reared in this farm. With the exception of the ocular sign, no systemic sign has been evident. The condition of the right eye showed no response to antibiotic treatment using cefazolin (Meiji Seika Pharma, Japan). The left eye appeared normal. Blood counts and serum biochemistry were within normal limits.

**Figure 2:**
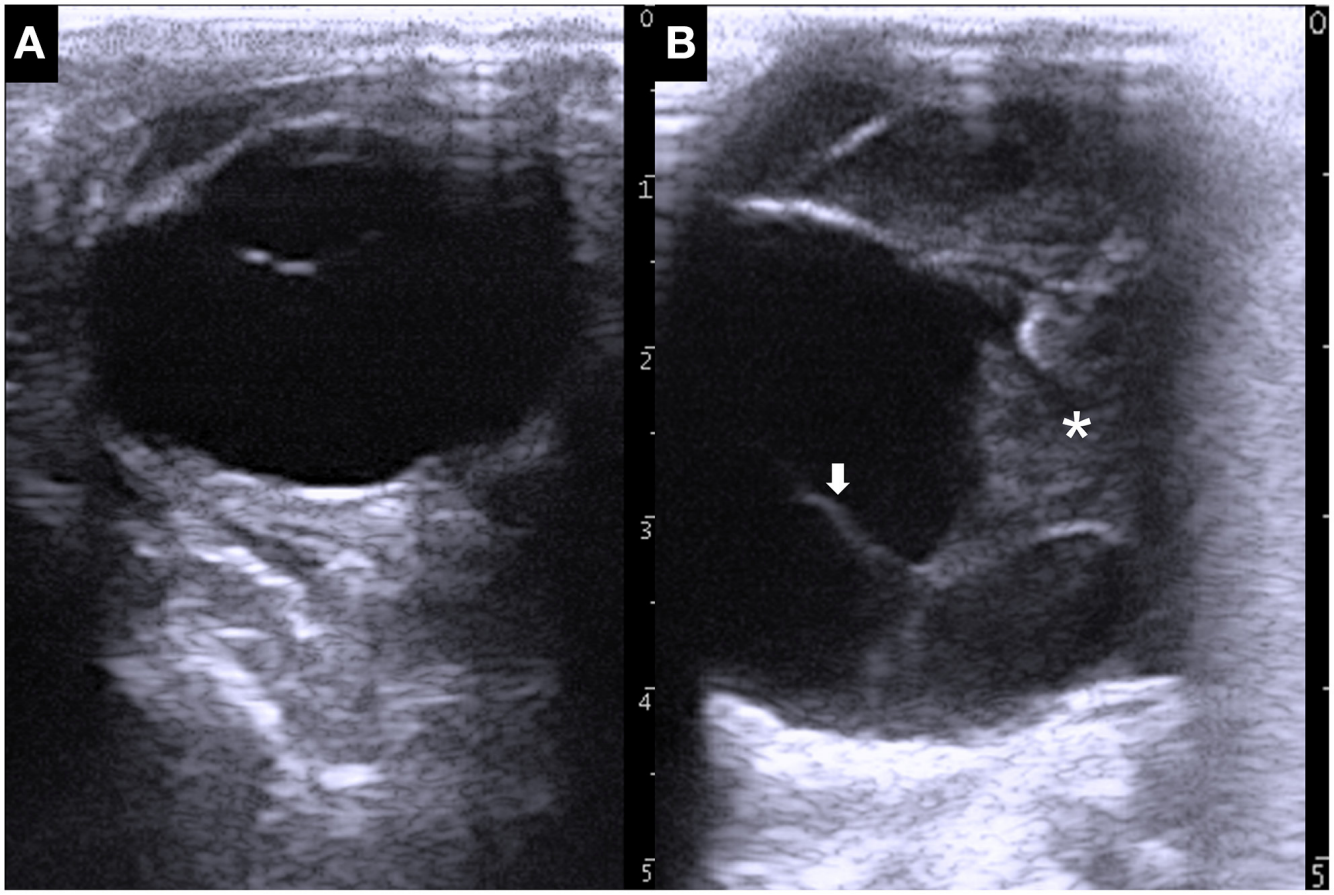
Ultrasonographic images of the left eye **(A)** and right eye **(B)**, showing the larger size of the right eyeball compared with that of the left eyeball. A 2-cm-thick echogenic parenchymal lesion (asterisk) is evident together with a floating membrane-like structure (arrow) in the anterior side of the right eyeball.

Ultrasonography of the eyes was performed, with the animal in a standing position, using a portable ultrasound device (MyLabOne VET, Esaote Corporation, Genova, Italy). Application of a 10.0 MHz linear transducer to the left eyeball revealed a normal structure comprising the echogenic cornea, the anechoic lens lined by the echogenic anterior and posterior lens capsules, and anechoic vitreous fluid inside the hyperechoic scleroretinal rim (Figure 2A). Ultrasonogram of the right eyeball, an ~2-cm-thick heterogenic echogenic lesion was evident in the anterior region (Figure 2B). The margin of the lesion appeared as an irregular hyperechoic line with a floating, membrane-like structure. Anechoic fluid was seen within the eyeball more distally than the echogenic lesion. The structure of the lens and the iris could not be identified within the right eyeball.

**Figure 3:**
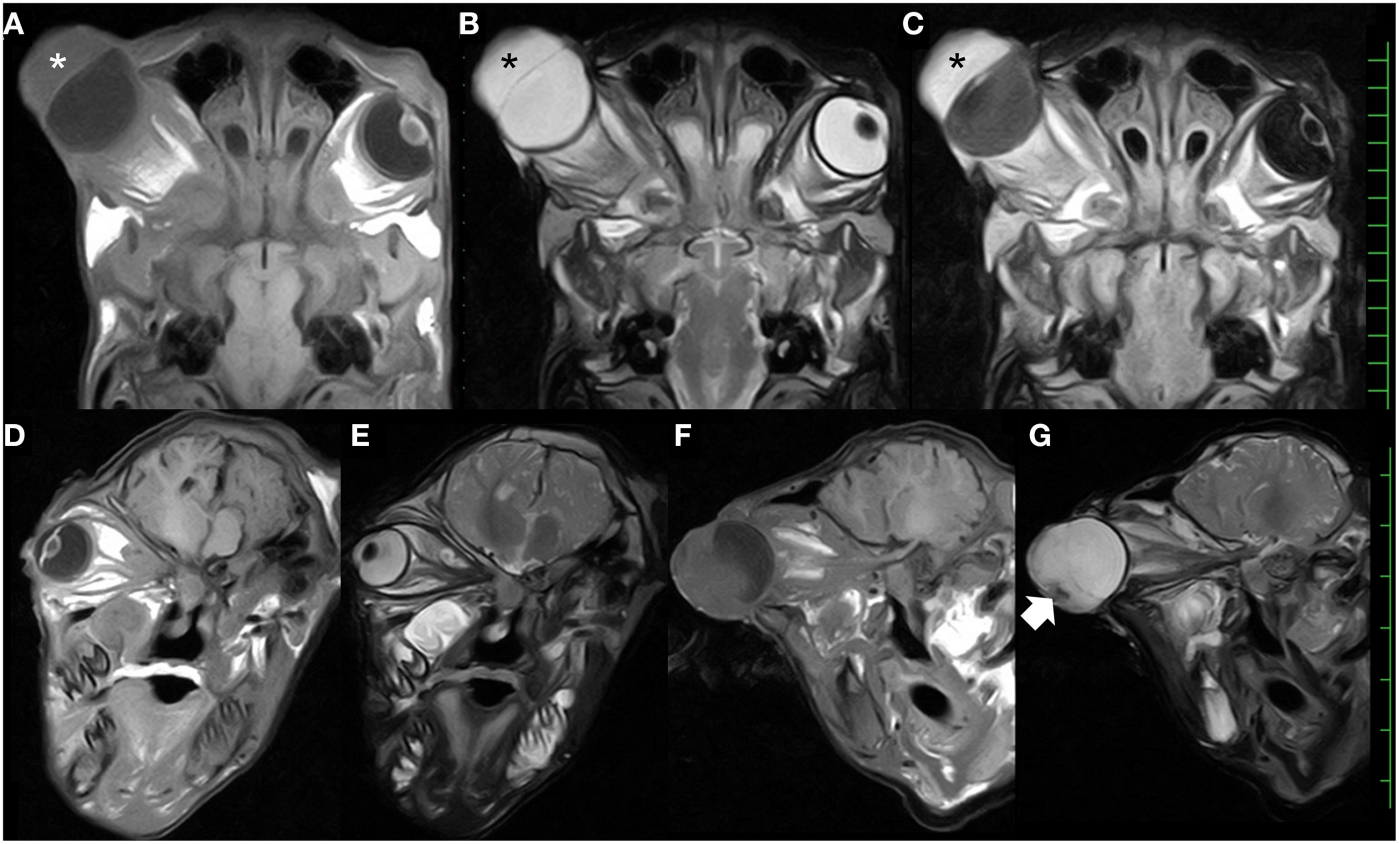
Dorsal T1-weighted **(A)**, T2-weighted **(B)**, and fluid-attenuated inversion recovery **(C)** magnetic resonance images, showing the presence of the thickened parenchymal lesion (asterisks) located in the anterior region of the enlarged eyeball. Oblique sagittal T1-weighted and T2-weighted magnetic resonance images of the left eye **(D,E)** and the right eye **(F,G)**, showing the optic nerves running within the retrobulbar region. The hypointense structure (arrow) is seen in the front and lower side of the hyperintense anterior lesion within the right eye. Scale: 10 mm.

MRI of the skull was performed using a low-field scanner (AIRIS Vento 0.3 T, Hitachi Medical Corporation, Tokyo, Japan) and a human knee coil. The animal was placed in left recumbency under general anesthesia with 0.2–0.3% isoflurane after sedation with 0.3 mg/kg xylazine hydrochloride. T1-weighted (time of repetition (TR), 450; time of echo (TE), 21; slice thickness, 5 mm), T2-weighted (TR, 3224; TE, 100; slice thickness, 5 mm), and fluid-attenuated inversion recovery (FLAIR; TR, 11000; TE, 100; slice thickness, 5 mm) images were acquired. In the dorsal MRIs, a half-moon-shaped lesion with a maximum thickness of 2.1 cm was present in the anterior region of the right eyeball (Figures 3A–C). The signal intensity of the lesion was homogeneously isointense compared with the peripheral soft tissues (the ocular muscles and brain structure) in T1-weighted images (Figure 3A); hyperintense (identical with those of the vitreous fluid) in T2-weighted images (Figure 3B); and hyperintense compared with the peripheral soft tissues and the vitreous fluid in FLAIR images (Figure 3C). In dorsal MRIs of the right eyeball, the structures of the cornea, the lens, and the ciliary body were not evident, although the lens was clearly imaged as mildly hypointense content inside the hyperintense membrane in the T1-weighted images of the left eyeball. In addition, the structure of the scleroretinal rim within the right eyeball was unclear compared with the smooth and hyperintense line within the left eyeball in T1-weighted and FLAIR images. In the T1-weighted and FLAIR images of the right eyeball, the vitreous fluid was hypointense, but the intensity was mildly increased compared with that in the image of the left eyeball. There were no differences in the hyperintensity of the vitreous fluid between the left and right eyeballs in T2-weighted images. The oblique sagittal MRIs were constructed parallel with the line passing from the origin of the optic nerve to the optic chiasm (Figures 3D–G). The hypointense structures of the optic nerve and the ocular muscles were clearly enhanced by the hyperintense peripheral structures, including the fat tissue, in the T1- and T2-weighted images. The optic nerve ran almost straight within the retrobulbar region and curved upward to enter the brain via the optic chiasm in the left eye (Figures 3D,E). In the right eye, no abnormalities of the optic nerve, such as swelling and curvature due to retrobulbar mass, were evident (Figures 3F,G). In the T2-weighted image, a hypointense structure was seen at the front and lower sides of the hyperintense anterior lesion of the right eyeball, although this was not evident in the T1-weighted image. The brain structure did not appear abnormal.

Soon after the MRI examination, ultrasound-guided centesis of the right vitreous cavity enabled the collection of a clear, yellowish fluid. A cytological examination revealed few cellular components, and bacteriological examination did not indicate the presence of any bacteria. These clinical findings were suggestive of anterior segment dysgenesis.

Three days after the examinations, enucleation of the right eye was performed. An incision was made along a 1-cm margin from the eyelid. The eyeball was easily separated from the peripheral soft structures with a deeper incision to the conjunctival surface. A 20-mm intraocular silicone prosthesis was placed into the empty socket following the removal of the right eye. The orbit was closed by simple sutures of the subcutaneous tissues and the skin. The animal was treated postoperatively by 1-week administration of cefazolin. One week after surgery, the surgical wound exhibited intact healing without suppuration, and there were no postoperative complications.

**Figure 4:**
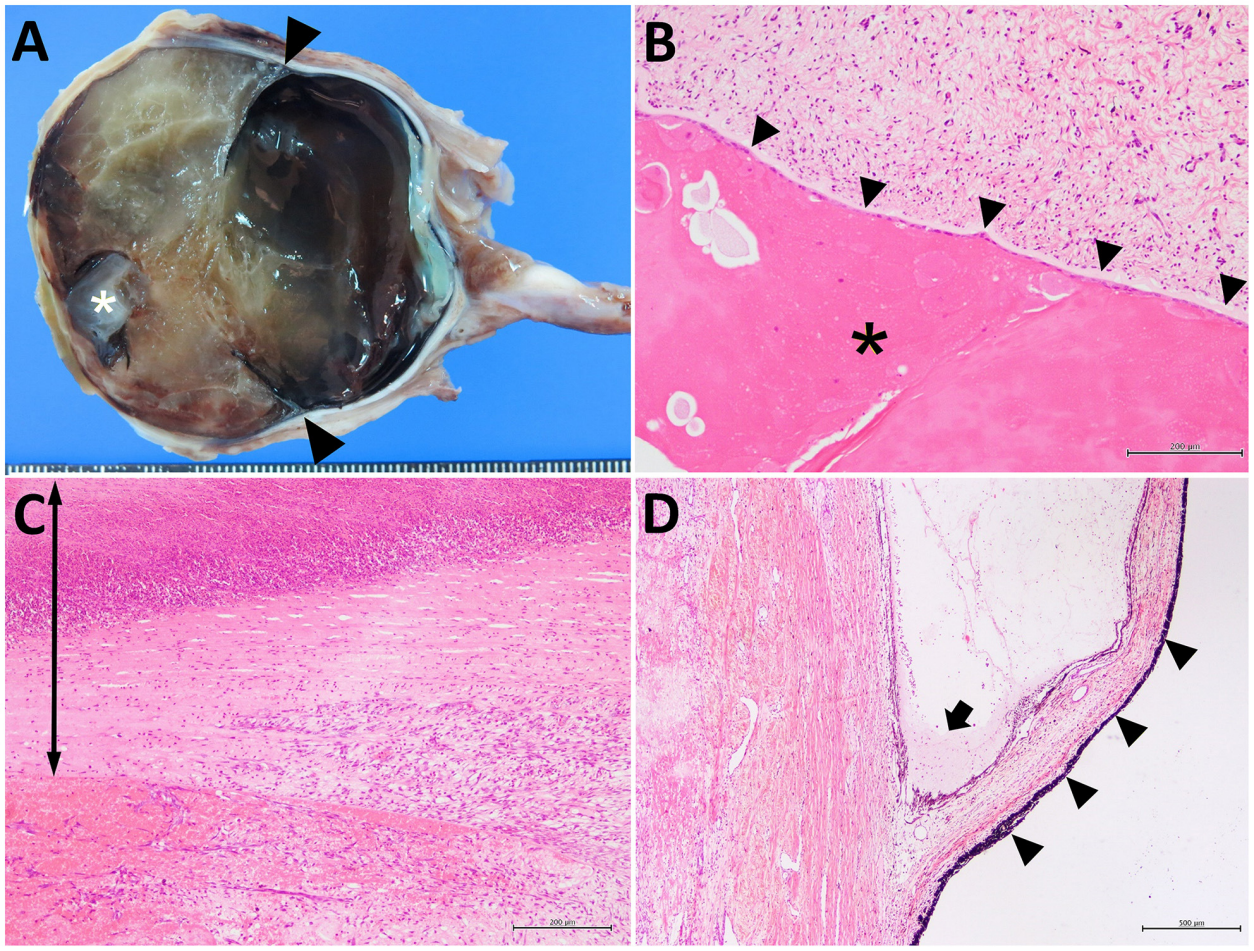
The cut surface of the affected right eye **(A)** and the histological sections of the lens-like structure **(B)**, the thickened anterior structure **(C)**, and the iris **(D)**. In **(A)** a 1-cm-diameter, whitish, globular lens structure (asterisk) is located on the subcornea. The structures of the iris are present (arrowheads). Scale bar = 1 mm. In **(B)**, marked degeneration of the lens (asterisk) is attached to the fibro-vascular tissues (arrowheads). In **(C)**, corneal tissue is thickened (arrow) and shows marked infiltration of neutrophils and fibroblastic cells (right-side). The surface is ulcerated and necrotic (upper region). The inner corneal epithelium cannot be identified. Hemorrhagic changes are also visible in the lower left-side of the photo (anterior chamber). In **(D)**, the atrophic iris (arrowheads) and ciliary body are seen. Exudate and some neutrophils are observed in the iridocorneal angle (arrow). **(B)** bar = 200 μm; **(C)** bar = 200 μm; **(D)** bar = 500 μm.

The removed right eyeball was markedly enlarged (67-mm diameter). The cut surface revealed an anterior chamber region containing soft, yellowish tissue and a round, whitish globe (the atrophied lens) (Figure 4A). A pair of valvular structures, consistent with the iris and ciliary bodies, was located in the center of the eyeball. The posterior region, including the vitreous body and the optic nerve, showed no significant changes. Microscopically, the lens showed marked degeneration with coagulation and cleaved substances (Figure 4B). The lens was located beneath the cornea, with bridging fibro-vascular tissues. The cornea was thickened by severe infiltration of neutrophils and showed signs of degeneration and necrosis (Figure 4C). Most of the surface epithelium of the cornea was ulcerated, and some bacterial colonies were noted. The inside layer of corneal epithelial cells and Desmet's membranes were not detectable; marked inflammation and hemorrhagic changes were evident. The anterior and posterior regions of the chamber were filled with fibrillar materials, neutrophils, erythrocytes, exudate, degenerated cell debris, and some pigmented cells. The iris was thin; however, its structure was conserved, including the iridocorneal angles and ciliary bodies (Figure 4D). A slight increase in the number and activation of glial cells was observed in the optic nerve bundles. Based on the histological findings, the present case was diagnosed as an anterior segment dysgenesis, concurrent with an infectious inflammation within the area of the anterior segments, associated with the dried, hard change in the anterior surface of the right eye.

## Discussion

The combination of ultrasonography and MRI used in the present case was able to show the characteristic appearance of an ASD-affected eyeball, resulting in the antemortem diagnosis of this condition. The imaging findings included aplasia of the lens, the thickened anterior segment of the eye, and enlargement of the eyeball due to the excess accumulation of vitreous fluid. These ocular abnormalities can be explained by the etiology of ASD. This condition occurs as a result of the abnormal maturation of neuro-ectodermal cells during the organogenetic period of the eye (18). In the early embryonic eye, complete separation of the lens vesicle from the surface ectoderm, which is the origin of the corneal epithelium and lens vesicle, triggers the migration of mesodermal tissue; this follows the formation of the corneal stroma, corneal endothelium, papillary membrane, ciliary body, and trabecular meshwork (2, 17, 19). Incomplete separation can result in structural adhesion between the cornea and the lens, leading to the complete or incomplete absence of the lens (referred to as “aphakia” and “microphakia”, respectively) at birth (2, 3, 19). In cases of bovine microphakia, a rudimentary lens, part of the lens capsule, and a dysplastic lens are commonly embedded within the sclera, the cornea, or the anterior uvea (3, 20). In addition, the abnormal migration of mesodermal tissues subsequent to the incomplete separation of the lens vesicle possibly contributes to malformation of the anterior segment of the eye, including the ciliary body and the iris (21). This malformation may affect the Schlemm's canal and trabecular meshwork drainage structures located at the anterior segment angle, where the iris and cornea meet. This disruption of drainage function is one possible reason for the elevated intraocular pressure seen in many human cases of ASD (21). Exophthalmos is the most progressive ocular sign associated with increased intraocular pressure and the flow of aqueous humor into the vitreous cavity and has frequently been observed in calves with ASD (4).

Ocular ultrasonography has previously been used to diagnosis Peters anomaly (a type of ASD) in a human patient, which manifested as a hyperechoic pattern in the anterior chamber of an eye that lacked a lens structure (22). Ocular ultrasonography has previously been applied in adult cattle, via the palpebral membranes, to observe healthy eyes when the animals were restrained in a crush or stocks, without requiring the use of sedation or perineural and/or topical analgesia (23). These previous trials succeeded in visualizing both the hyperechoic structures (including the cornea, the anterior lens capsule, the posterior lens capsule, and the scleroretinal rim) and the anechoic structures (including the aqueous humor, the lens, and the vitreous humor) (23). In addition, the iris adjacent to the anterior lens capsule, and the ciliary body, could be seen as moderately echogenic structures (23). When using ultrasonography to make a differential diagnosis between ASD and other ocular diseases, the cornea, the anterior and posterior chambers, the lens, the iris, and the ciliary body are the significant ocular structures that should be evaluated. Based on morphological abnormalities of these structures, such as microphthalmia, hypoplasia, and/or dysplasia of the lens, the use of ultrasonography in adult cattle has enabled the antemortem diagnosis of congenital ocular diseases, although the images have unfortunately not been shown (17). In the majority of bovine ocular diseases, the morphological abnormalities of the eyeballs result in abnormal rotation or dislocation of the eyeballs, leading to exophthalmos. Various types of ophthalmoparesis and prolapse of the eyeball are associated with palpebral paralysis due to physical compression from cranial masses, cavernous sinus syndrome, or BCSE; all are common causes of abnormal rotation of the eyeball (10, 11, 13). BLV-induced retrobulbar masses can injure the central nervous system as well as causing direct pressure on the eye, causing the eyeball to protrude (6, 7). Theileriosis also increases the pressure in the orbits (8). These conditions may cause the eye to enlarge but generally do not alter the original shape of the anterior segment of the eye. An atrophic iris and the ciliary body could not be visualized on the ultrasonogram in the present case. This may possibly be because newborn animals have smaller ocular structures than adult animals (23).

Retrobulbar lesions, another major cause of exophthalmoses (6, 7), may be visualized from the clinical use of ultrasonography in small animal practice (15). Retrobulbar lesions are suspected if the retrobulbar structures and the eye-socket wall show an altered shape and echogenicity (16). Retrobulbar abscesses and lymphomas often appear as oval hypoechoic structures (15, 16). However, attenuation of ultrasonic waves and visual interference caused by bone may make ultrasonographic visualization of lesions derived from outside of the eye socket difficult (10–12). Ultrasound biomicroscopy is a non-invasive imaging technique for the high-resolution evaluation of anatomical features of the anterior segment of the eye; this may be of great benefit for diagnosing ASD and assessing the underlying mechanism of exophthalmos in cattle (18).

MRI is a superior imaging modality compared with ultrasonography and CT, providing better contrast between different intraocular tissues (16). On MRI of the eye, the lens is an easily distinguishable structure, based on its different signal intensity compared with that of vitreous humor, in T1-weighted, T2-weighted, and FLAIR images, and the well-defined, hyperintense contours of the anterior and posterior lens capsules in T1-weighted and FLAIR images (16). This corresponds with the applicability of MRI for the diagnosis of ASDs, which commonly involve abnormalities of the anterior segments of the eye (2–4, 21, 22). In the present case, when the anterior segments of the eye were compared between the left (healthy) and right (affected) eyes on the same dorsal plane, the morphological abnormalities comprised a thickened anterior part of the eyeball and an absence of the lens structure. This strongly indicated that the parenchymal lesion was not a simple swelling of the cornea, such as that caused by infectious keratitis. The MRI finding of incomplete absence of the lens (microphakia) was significant evidence relating to the antemortem suspicion of congenital ocular disease and complemented the ultrasonographic findings, although aphakia was suspected based on ultrasonography. In the present case, excess accumulation of fluid in the vitreous cavity of the right eye was also estimated by the FLAIR image findings, with the vitreous humor appearing at a slightly higher signal intensity than that of the healthy vitreous humor in the left eye. The different signal intensity of the right vitreous humor suggested intraocular infection or increased cellular components within the vitreous humor, allowing recognition of the need for the subsequent cytological and bacteriological examinations. In the present case, the comprehensive clinical diagnosis including use of ultrasonography and MRI could contribute to exclusion of ocular infection such as endophthalmitis (24), providing the significant evidence for judgement of surgical intervention. Thus, MRI is commonly useful in clarification of the detailed pathological changes in each ocular structure, through the combined evaluation of multiple images obtained from various sequences (16). This property of MRI is useful for therapeutic decision-making and preoperative planning.

MRI can provide a good morphological assessment of retrobulbar structures, such as the optic nerve and the optic chiasm, as well as the deeper brain structure, allowing the reliable determination of the extent of disease within or beyond the eye socket, using oblique sagittal images (16). This advantage suggests that MRI is equally as useful as CT and superior to ultrasonography (15, 16). MRI and CT have previously been used to clarify cavernous sinus syndrome secondary with multiple formations of abscess within the skull or invasion of the nasal osteoma toward the eye in the affected cattle (10, 12). In MRI, the good contrast shown by the retrobulbar region may be helpful for diagnosing retrobulbar lesions, such as retrobulbar lymphomas, which can invade and spread through the ocular muscles and surround the optic nerve in cattle (7). Additionally, MRI can allow visualization of brain diseases, as the rare concurrent involvements of bovine hereditary ocular defects (1). On the other hand, MRI may be inferior to CT for visualization when destructive and osteolytic changes of the orbit, associated with invasion of the primary lesions, have occurred (16).

Enucleation is necessary for an enlarged eyeball that prevents closure of the eyelid. Not being able to close the eyelid can lead to keratitis, subsequent perforation, and panophthalmitis (2, 3). However, the need for enucleation should be assessed based on the cause of the enlarged eyeball. Enucleation is recommended for ASD-induced enlargement of the eyeball, because it enables surgical removal of the intraocular cause. On the other hand, enucleation may not be recommended if compression from lesions located in the retrobulbar region or outside the eye socket is suspected, because enucleation does not completely remove the underlying cause in such cases. Imaging is therefore very helpful for making therapeutic decisions.

## Conclusion

Ultrasonography should be used as the first choice for the diagnosis of ASD in bovine practice, because of its convenience and the avoidance of chemical restraint (16). More importantly, an ultrasonographic finding of a thickened anterior segment, in which no lens structure is evident, is suggestive of ASD (22). In addition, ultrasonography is highly applicable for imaging-assisted centesis and biopsies because it facilitates real-time observation (16). However, ultrasonography is inferior to MRI in the quality of the intraocular image, as hypoplasia of the lens is not ultrasonographically evident in this case. MRI is complementary to a ultrasonographic evaluation, based on the higher quality intraocular images, allowing clear visualization of the hypoplastic lens and retrobulbar structures, although sedation or anesthesia are required for examination with MRI (16).

## Data Availability Statement

The raw data supporting the conclusions of this article will be made available by the authors, without undue reservation.

## Ethics Statement

The animal study was reviewed and approved by Tottori University Regulations on Animal Experiments. Written informed consent was obtained from the owners for the participation of their animals in this study.

## Author Contributions

TT supervised both diagnosis and therapy for the present case, reviewed the literature, and prepared the manuscript. YO, TA, YM, MY, and TO performed surgery. YS and TM performed pathological examination. NI and TI performed the imaging examinations. All authors read and approved the final manuscript.

## Conflict of Interest

The authors declare that the research was conducted in the absence of any commercial or financial relationships that could be construed as a potential conflict of interest.

## Publisher's Note

All claims expressed in this article are solely those of the authors and do not necessarily represent those of their affiliated organizations, or those of the publisher, the editors and the reviewers. Any product that may be evaluated in this article, or claim that may be made by its manufacturer, is not guaranteed or endorsed by the publisher.
